# Time-resolved x-ray crystallography capture of a slow reaction tetrahydrofolate intermediate

**DOI:** 10.1063/1.5086436

**Published:** 2019-03-01

**Authors:** Hongnan Cao, Jeffrey Skolnick

**Affiliations:** Center for the Study of Systems Biology, School of Biological Sciences, Georgia Institute of Technology, 950 Atlantic Drive, NW, Atlanta, Georgia 30332, USA

## Abstract

Time-resolved crystallography is a powerful technique to elucidate molecular mechanisms at both spatial (angstroms) and temporal (picoseconds to seconds) resolutions. We recently discovered an unusually slow reaction at room temperature that occurs on the order of days: the *in crystalline* reverse oxidative decay of the chemically labile (6S)-5,6,7,8-tetrahydrofolate in complex with its producing enzyme *Escherichia coli* dihydrofolate reductase. Here, we report the critical analysis of a representative dataset at an intermediate reaction time point. A quinonoid-like intermediate state lying between tetrahydrofolate and dihydrofolate features a near coplanar geometry of the bicyclic pterin moiety, and a tetrahedral *sp*^3^ C6 geometry is proposed based on the apparent mFo-DFc omit electron densities of the ligand. The presence of this intermediate is strongly supported by Bayesian difference refinement. Isomorphous Fo-Fo difference map and multi-state refinement analyses suggest the presence of end-state ligand populations as well, although the putative intermediate state is likely the most populated. A similar quinonoid intermediate previously proposed to transiently exist during the oxidation of tetrahydrofolate was confirmed by polarography and UV-vis spectroscopy to be relatively stable in the oxidation of its close analog tetrahydropterin. We postulate that the constraints on the ligand imposed by the interactions with the protein environment might be the origin of the slow reaction observed by time-resolved crystallography.

## I. INTRODUCTION

Time-resolved crystallography is an experimental technique that can detect molecular changes at atomic and temporal resolutions.^1^ Due to the feasibility of rapid reaction initiation by a laser pulse, this technique has been widely used to study light-active protein systems including myoglobin,^2^ hemoglobin,^3^ photoactive yellow protein,^4–6^ photosystem II,^7–9^ and rhodopsin.^10^ Recent advances in sample delivery systems and femtosecond X-ray free electron lasers (XFELs) have allowed time-resolved serial femtosecond crystallography (TR-SFX) to be extended to other systems as well.^1,11–13^ For example, the mix-and-inject method can rapidly and uniformly initiate an enzymatic reaction^11,12^ or an RNA-ligand interaction^13^ in micro/nanocrystals before diffraction whose rate is limited by diffusion. To the best of our knowledge, time-resolved crystallography has almost exclusively been applied on time scales of picoseconds to seconds. Here, using traditional cryocrystallography at a synchrotron source without rapid mixing or use of a laser pulse, we recently discovered the slow oxidative decay of tetrahydrofolate to dihydrofolate in the enzyme bound crystalline form at room temperature, with the critical transition occurring 2–3 days after the crystallization setup.^14^ We present an analysis of the third day's crystal structure to estimate the putative intermediate's geometry and population relative to dihydrofolate and tetrahydrofolate. The implications of the current observation on the molecular mechanism of tetrahydrofolate to dihydrofolate conversion and its possible generalization to other systems are discussed.

## II. MATERIALS AND METHODS

### A. Protein expression and purification

C-terminal 6xHis-tagged *Escherichia coli* DHFR (dihydrofolate reductase, with 100% sequence identity to UNIPROT sequence sp|P0ABQ4| or sp|P0ABQ5|) was generously provided by Drs. Eugene Shakhnovich and João Rodrigues from Harvard University. DHFR was overexpressed in *E. coli* BL21 and then purified by Ni-NTA and size exclusion chromatography as previously described.^15^ The initial protein stock was stored at −80 °C at a concentration of 30 mg ml**^−^**^1^ in 20 mM Tris, at pH 8, and in 1 mM dithiothreitol (DTT). Polyethylene glycol 3350 was purchased from Hampton Research. All other chemicals and reagents were obtained at the highest quality available from Sigma-Aldrich or ThermoFisher and used without further purification.

### B. Protein crystallization

The current intermediate time point complex was obtained using the same crystal growth condition as in our previously reported binary tetrahydrofolate complex (PDB: 6CW7).^14^ Briefly, as-purified eDHFR was crystallized by sitting drop vapor diffusion using a 1:1 v/v mixing of 20 mg ml^−1^ DHFR solution in 13.3 mM Tris at pH 8, 16.7 mM HEPES at pH 7.3, 33.3 mM NaCl, and 0.67 mM DTT with the reservoir solution containing 0.1 M MES, at pH 6.5, with 30% w/v PEG 3350, and 0.4 M MgCl_2_. Mixed drops of 0.8 *μ*l were equilibrated over a reservoir solution of 50 *μ*l on a MRC 2-well plate (Hampton Research) and incubated at 20 °C in the dark. A single crystal per dataset was harvested at 3 days after setting up the crystallization drops (as opposed to 2 days for the tetrahydrofolate complex or 2 weeks for the dihydrofolate complex in our earlier report)^14^ and then cryoprotected with LV CryoOil and flash-frozen in liquid N_2_.

### C. Data collection and refinement

Diffraction data were collected at the Advanced Photon Source at Argonne National Laboratory on the LRL-CAT (31-ID-D) beamline at 100 K. The detector was a Rayonix 225 HE CCD (Rayonix) using a single wavelength of 0.97931 Å. The intermediate time point (3 days of crystal growth) dataset was collected and processed to a resolution of 1.35 Å. The datasets were indexed, integrated, and scaled using XDS.^16^ The structure was determined by molecular replacement with Phaser_MR^17^ (using the protein coordinates of the tetrahydrofolate complex as the search model, PDB ID: 6CW7) and completed by alternating rounds of manual model building with COOT^18^ and both reciprocal and real space refinements using phenix.refine of the PHENIX suite.^19^ The ligand was originally built into the model as tetrahydrofolate (PDB Ligand ID: THG) based on the apparent tetrahedral geometry suggested by the clear omit electron density. Met16 is very close in geometry to that in the tetrahydrofolate complex. We conjecture that it acts as a clamp that is partly responsible for the slowdown of tetrahydrofolate to dihydrofolate oxidation. The Met20 loop appears to be partially disordered for residues Glu17-Asn18-Ala19 in contrast to the clearly traceable conformations of the end-state complexes with tetrahydrofolate (PDB: 6CW7) or dihydrofolate (PDB: 6CXK) that were visible in our earlier report.^14^ On the other hand, Met20 and Pro21 are clearly visible and are quite close to their conformation in the dihydrofolate complex. Thus, the residues in the Met20 loop adopt structure of the intermediate in complex with DHFR are a hybrid of the two endpoint conformations with disorder in the middle residues of the loop. Mg^2+^, Cl^−1^ ions, and water molecules were added to the model after the ligand was built in.

The fully refined model contains a ligand geometry containing coplanar pterin rings and a tetrahedral C6 geometry suggesting a possible intermediate state. This contrasts with the puckered pterin previously observed for the tetrahydrofolate complex despite the same ligand restraints being used.^14^ Due to the uncertainty of the exact chemical structure and fractional population of the ligand, we further performed refinement analysis by comparing one-state (a single intermediate), two-state (a linear combination of two end states, dihydrofolate and tetrahydrofolate), three-state (a linear combination of both end states and the proposed intermediate) models. Briefly, the previously reported end-state models (PDB IDs: 6CW7 and 6CXK) were each aligned to the current protein model and their ligand coordinates extracted and merged into the current model with COOT.^18^ To achieve the linear combination of ligand states, only the occupancy and individual B-factors of the structures were refined in reciprocal space with their coordinates kept intact. The initial ligand occupancy was assigned as 0.5 to each ligand for the two-state model. In the three-state model, the initial ligand occupancy was intentionally assigned as 0.1 to the proposed intermediate state and 0.45 to each of the two end-state ligands to test how these three states compete with each other in terms of percentage population in the current refinement scheme. The final refined occupancy values of the three ligands appear to be insensitive to their initial occupancy assignments (e.g., equal initial occupancy of 0.33 for each ligand state) and always converged to the same results, i.e., the intermediate state is the most populated at 0.45 which approximately equals the combined occupancy of two-end states. The structures determined in this study display Ramachandran statistics absent of outliers, with 98.8% of the residues in the most favored regions and 1.2% of residues in additionally allowed regions of the Ramachandran diagram defined by MolProbity^20^ (Table I). All structures are displayed using PyMOL^21^ unless otherwise stated. The coordinates and reflection files of the structures are deposited in the Protein Data Bank (www.rcsb.org) under PDB IDs: 6MR9 (one-state), 6MT8 (two-state), and 6MTH (three-state).

**TABLE I.** Statistics for X-ray data collection and structural refinement. Values in parentheses are for the highest resolution shell.

Statistic	One intermediate	Two end states	Three states	Bayesian difference^j^
Protein Data Bank ID code	6MR9	6MT8	6MTH	
Spacegroup	P2_1_2_1_2_1_			P2_1_2_1_2_1_
Cell dimensions				
*a, b, c* (Å)	34.1, 51.7, 79.0			33.9, 51.5, 77.8
*α, β, γ* (^o^)	90.0, 90.0, 90.0			90.0, 90.0, 90.0
Wavelength (Å)	0.97931			0.97931
Resolution of data collection (Å)	31.3–1.35 (1.40–1.35)			31.1 – 1.35 (1.40-1.35)
No. of unique reflections	30678 (2550)			29117 (2419)
Completeness % (Å)	97.7 (82.7)			94.9 (80.1)
Redundancy	6.4 (3.1)			
*R*_sym_^a^	0.098 (1.369)			
*I*/*σ*^b^	10.8 (0.9)			4.3^b^
CC_1/2_^c^	0.997 (0.289)			
CC^*^^c^	0.999 (0.670)			
Resolution range in refinement (Å)	31.3–1.35 (1.40–1.35)			31.1–1.35 (1.40–1.35)
No. of unique reflections (total/test)	30673/3667			29113/2000
*R*_work_^d^ (%)	16.78 (35.54)	16.72 (35.46)	16.72 (35.47)	20.69 (36.00)
*R*_free_^e^ (%)	19.05 (35.18)	18.96 (35.17)	19.02 (35.20)	20.58 (34.13)
*R*_Bayes,work_^d^ (%)				11.47 (31.67)
*R*_Bayes,free_^e^ (%)				13.57 (32.26)
Mean coordinate error^f^ (Å)	0.17	0.19	0.17	0.15
Rmsd bond length (Å)	0.007	0.017	0.020	0.010
Rmsd bond angles (^o^)	1.23	1.36	1.42	1.38
Average B value (Å^2^) (overall/protein/waters/ligand)	20.8/18.9/32.5/28.4	21.1/19.1/32.4/26.0	21.2/19.2/32.5/26.1	19.3/17.5/30.6/26.9
No. of non-hydrogen atoms	1596	1628	1660	1672
No. of protein atoms	1357	1357	1357	1428
No. of waters	200	200	200	205
No. of ligand atoms	39	71	103	39
Ramachandran Statistics^g^ (%)	98.8, 1.2, 0	98.8, 1.2, 0	99, 1.2, 0	98, 1.4, 0.6
Individual ligand B-factor (Å^2^) (Intermediate/FH4/FH2)	28	22/21	23/23/23	25
Individual ligand occupancy (Intermediate/FH4/FH2)	1.0	0.47/0.45	0.47/0.21/0.22	1.0
RSCC^h^ (Intermediate/FH4/FH2)	0.94	0.95/0.95	0.95/0.95/0.95	0.97
RSR^i^ (Intermediate/FH4/FH2)	0.14	0.12/0.11,	0.10/0.11/0.11	0.05

^a^ *R*_sym_ = ∑_*hkl*_∑_*i*_ |*I_i_*(*hkl*) - ⟨*I*(*hkl*)⟩|/∑_*hkl*_∑_*i*_ *I*_*i*_(*hkl*), where *I_i_*(*hkl*) is the intensity of an individual measurement of the symmetry related reflection, and ⟨*I*(*hkl*)⟩ is the mean intensity of the symmetry related reflections.

^b^ *I*/*σ* is defined as the ratio of the averaged value of the intensity to its standard deviation. For Bayesian difference refinement, because the pseudo dataset contains only amplitude rather than intensity information, an overall *I*/*σ* is estimated by running the SFCHECK^34^ program of the CCP4 suite.^35^ 100% of the reflections in the pseudo dataset show *F*/σ(*F*) >2 using amplitude units.

^c^ CC_1/2_ = percentage of correlation between intensities from random half-datasets. CC_1/2_ above 0.1 is considered significant.^36^ CC^*^=[2CC_1/2_/(1+ CC_1/2_)]^1/2^. CC^*^ estimates the value of CC_true_. CC^*^ (or CC_1/2_) is a robust, statistically informative quantity useful for defining the high-resolution cutoff of diffraction data to improve the model quality.^36^.

^d^ *R*_work_ = ∑_*hkl*_ǁ*F*_obs_| - |*F*_calc_ǁ/∑_*hkl*_|*F*_obs_|, where *F*_obs_ and *F*_calc_ are the observed and calculated structure-factor amplitudes for the reflections being refined against. *R*_Bayes,work_ and *R*_work_ were calculated the same way except that the corresponding structure factors being used are *F*_diff_ of the pseudo dataset and *F*_obs_ of the experimental dataset, respectively.

^e^ *R*_free_ was calculated as *R*_work_ using randomly selected small fractions (typically <10%) of the unique reflections that were omitted from the structure refinement. *R*_Bayes,free_ was calculated the same way as *R*_free_ except that *F*_diff_ of an omit pseudo set was used.

^f^ Mean coordinate error was calculated based on maximum likelihood.

^g^ Ramachandran statistics indicate the percentage of residues in the most favored, additionally allowed and outlier regions of the Ramachandran diagram as defined by MolProbity.^19^

^h^ RSCC = ∑(*ρ*_obs_ – ⟨*ρ*_obs_⟩)(*ρ*_calc_ – ⟨*ρ*_calc_⟩)/[∑(*ρ*_obs_ – ⟨*ρ*_obs_⟩)^2^ ∑(*ρ*_calc_ – ⟨*ρ*_calc_⟩)^2^]^1/2^, where *ρ*_obs_ and *ρ*_calc_ are the observed and calculated electron densities, and ⟨*ρ*_obs_⟩ and ⟨*ρ*_calc_⟩ are the mean values of *ρ*_obs_ and *ρ*_calc_, respectively. RSCC is an abbreviation for the real-space correlation coefficient.

^i^ RSR = ∑|*ρ*_obs_ – *ρ*_calc_|/∑|*ρ*_obs_ + *ρ*_calc_|, where *ρ*_obs_ and *ρ*_calc_ are the observed and calculated electron densities, respectively. RSR is an abbreviation for the real-space R factor.

^j^ Bayesian difference refinement uses a pseudo dataset as described in the methods. Parameters such as Rsym are unavailable.

### D. Fo-Fo difference map

Fo-Fo difference electron density maps were calculated using the “Isomorphous Difference Map” utility of PHENIX.^19^ The input files are the coordinate (.pdb) files and structure factor (.mtz) files of the corresponding states being compared.

### E. Bayesian difference refinement

Bayesian difference refinement was performed using the default protocol developed by Terwilliger and Berendzen.^22^ This is a sensitive method to detect small but finite structural changes between two very similar protein structural models obtained from two very similar experimental X-ray diffraction datasets. It was reported to perform better or at least as good as individual refinements in estimating the finite atomic shifts (RMS of shifts), depending on the correlation coefficient of model errors between test datasets.^22^ The more similar the two datasets (hence the models) are, the more useful is the Bayesian difference refinement method.^22^ Briefly, a pseudo variant dataset with amplitudes and weights was generated by running FDIFF scripts in the SOLVE^23^ program based on *F*o (the observed amplitude of the structure factor) and *F*c (the amplitude calculated from the model) from both native and variant datasets. In our case, the native dataset was the tetrahydrofolate complex (PDB: 6CW7),^14^ and the variant dataset was the intermediate time point dataset. Both are isomorphous crystals with nearly identical unit cell parameters grown under the same crystallization conditions, with around a 24 h delay in harvesting (2 days vs. 3 days of crystal growth since setup).

The parameters of Bayesian difference refinement were defined originally by Terwilliger and Berendzen^22^ as follows

The value of *F*_diff_ is given by

$$F_{\text{diff}}=F_{c,\text{native}}+\beta *\;\left(F_{o,\text{variant}}-F_{o,\text{native}}\right).$$ (1)

The factor *β,* essentially the correlation coefficient between *F*o_native_–*F*c_native_ and *F*o_variant_–*F*c_variant_, is given by

$$\beta =E^{2}/\left(E^{2}+{A_{\mathrm{native}}}^{2}+{\sigma_{\mathrm{native}}}^{2}\right).$$ (2)

The weighting factor or pseudo experimental errors of the pseudo dataset is given by

$${\sigma_{\text{Fdiff}}}^{2}={\sigma_{\mathrm{variant}}}^{2}+{A_{\mathrm{variant}}}^{2}+1/\left[1/\left({{\sigma_{\mathrm{native}}}^{2}}^{+}{A_{\mathrm{native}}}^{2}\right)+1/E^{2}\right].$$ (3)

*E*^2^ represents the total correlated model error vs. the data between the native and variant datasets. *A_native_*^2^ and *A_variant_*^2^ represent uncorrelated model errors of the native and variant datasets, respectively. *σ_native_* ^2^ and *σ_variant_* ^2^ represent the experimental measurement errors of the native and variant datasets, respectively. Both *β* and *σ*_Fdiff_^2^ terms contain certain weighted information derived from errors in both models and the experimental measurement of the two original datasets. The pseudo dataset containing information of *F*_diff_ (amplitudes) and *σ*_Fdiff_^2^ (weights) can then be used as input for the structure factor data in phenix.refine following regular refinement procedures.

## III. RESULTS AND DISCUSSION

### A. Structural refinement using an individual dataset

**FIG. 1.**
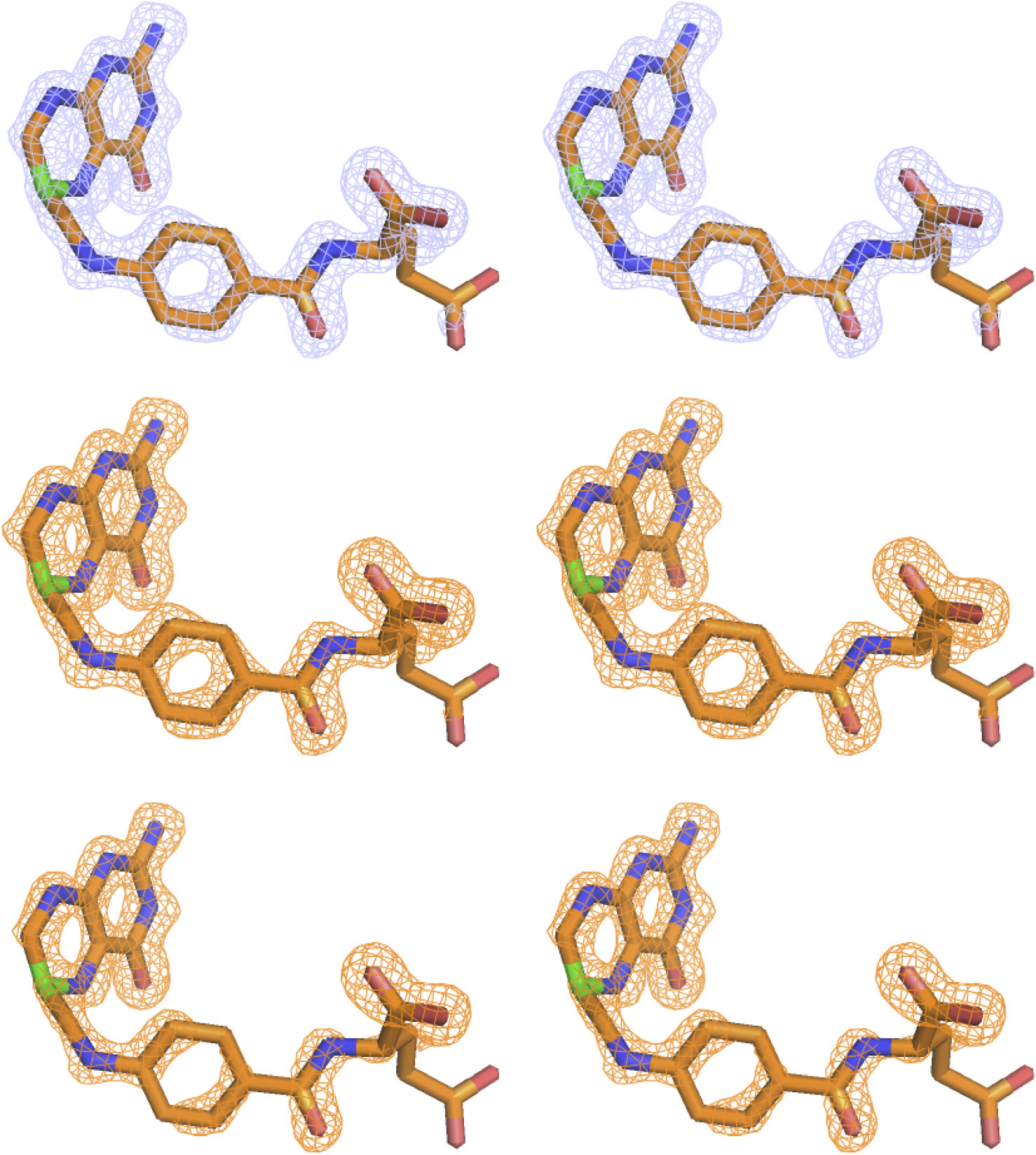
The omit electron densities of the putative intermediate during tetrahydrofolate reverse oxidation in stereo views. Top panel, 2mFo-DFc omit map at 1 σ; middle panel, mFo-DFc omit map at 3 σ; bottom panel, mFo-DFc omit map at 4 σ. Nitrogen in blue, oxygen in red, carbon in orange except C6 in green. Hydrogen atoms are not shown for simplicity. The left and right images are stereo views.

**FIG. 2.**
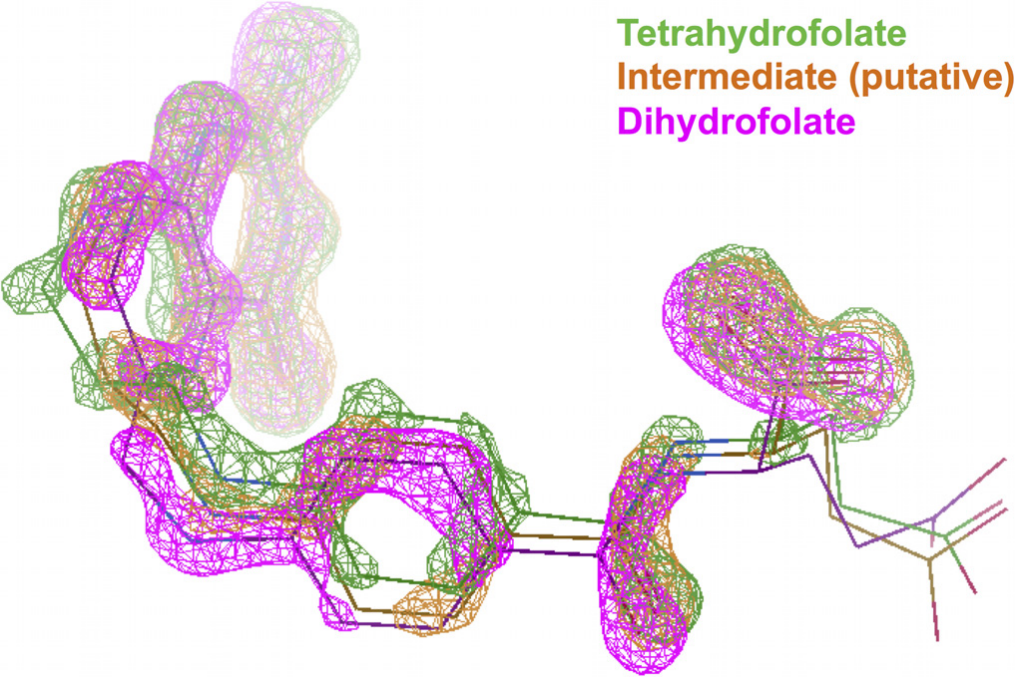
Overlay of omit electron densities of three different datasets based on the protein structural alignment. The superposition is achieved by the “transform map by LSQ model fit” function in COOT. The absolute electron density level at 0.65 e Å^−3^ was used as cutoff. Time-resolved electron density changes can be visualized. The electron densities are shown as meshes, and the corresponding ligands are shown as thin sticks to guide the view. The color codes are tetrahydrofolate in green, the putative intermediate in orange, and dihydrofolate in magenta.

**FIG. 3.**
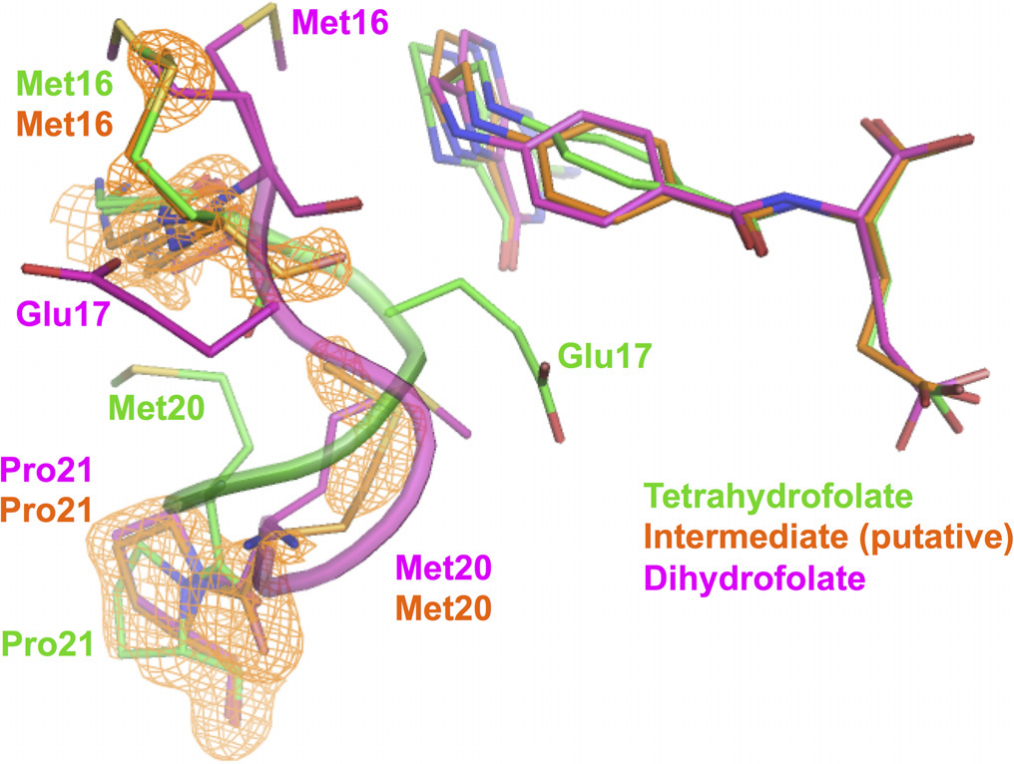
Coupled motion of the Met20 loop along with tetrahydrofolate oxidation. The 2mFo-DFc map at 1 σ omitting the ligand is calculated based on the intermediate dataset and shown for the Met20 loop. The residues Glu17-Asn18-Ala19 are disordered in the intermediate state structure with little electron density unlike the two end states that are shown as cartoons. The residues at the two anchors of the loop show densities that resemble different end states tetrahydrofolate and dihydrofolate complexes, respectively. Color codes for carbon are tetrahydrofolate in green, intermediate in orange, and dihydrofolate in magenta.

We first performed a regular refinement on the intermediate time point dataset (3 days of crystal growth). The details of the structural determination using molecular replacement and subsequent refinement are described in Sec. II. Both the 2mFo-DFc and mFo-DFc omit electron density maps indicate that the bound ligand retains an *sp*^3^ C6 with a tetrahedral geometry (Fig. 1). When the structure is refined with a single ligand [using PDB Ligand THG, (6S)-5,6,7,8-tetrahydrofolate], the pterin rings of the ligand appear to be near coplanar as compared to the puckered rings in the tetrahydrofolate bound complex. Hence, the ligand density suggests a putative intermediate state, which not only chemically resembles tetrahydrofolate in the *sp*^3^ C6 hybridization state (as opposed to the *sp*^2^ C6 of 7,8-dihydrofolate) but also partially mimics dihydrofolate by its nearly flattened pterin. The overall direction of the changes of the ligand geometry during the decay of tetrahydrofolate in the enzyme complex could be visualized from the ligand electron density changes based on the superposition of the corresponding models at different time points using COOT^18^ (Fig. 2). The putative intermediate state displays omit ligand electron densities in between those of the two end states. This is consistent with our previous report on the concomitant C6 *sp*^3^ to *sp*^2^ transition and the rotation of the benzoyl ring of the ligand during the decay of the binary complex of *E. coli* DHFR with tetrahydrofolate.^14^ In addition, the Met20 loop is partially disordered in the putative intermediate complex structure with little electron density for residues Glu17-Asn18-Ala19. Interestingly, in the putative intermediate complex, one anchor of the loop (Gly15-Met16) resembles the initial state tetrahydrofolate complex, and the other anchor (Met20-Pro21) is similar to the dihydrofolate complex at the completion of the decay (Fig. 3) Thus, it appears that both the ligand and Met20 loop conformations in the proposed intermediate state have distinct features that may represent a quasi-stable state captured during the slow reverse oxidative decay of the tetrahydrofolate complex. These mixed state results also suggest that the intermediate is real and not the result of averaging of the two endpoint conformations and implies that the time-resolved ligand and protein conformational changes are finitely coupled in the crystalline state of the tetrahydrofolate bound complex. These results corroborate previous NMR studies of *E. coli* DHFR on the mechanism of conformational selection in response to the identity of the bound ligand.^2^

We next refined the structure with mixed ligand states to estimate the population percentage since it is rare to observe a true intermediate state in time-resolved crystallography experiments.^1–13^ In particular, a linear combination of two end states (tetrahydrofolate and dihydrofolate) and all three states (tetrahydrofolate, putative intermediate, and dihydrofolate) was used to define the bound ligands with equal partial initial occupancy summed to 1. Since the initial and final states were well defined in a previous study, the atomic B factors and occupancy values were optimized with their coordinates intact. The refinement statistics are listed in Table I. It appears that in the reciprocal space as reflected by *R*_work_ and *R*_free_, both single state and multiple states yield similar statistics with the two-state model performing slightly better. The real-space correlation coefficient (RSCC) values of the individual ligands are comparable among the single intermediate state (0.94), two-state (0.95), and three-state (0.95) models (Table I). The individual ligand real-space R factors (RSR) and B-factors of the multi-state models appear to be relatively more favorable than the one-state model (Table I). In the three-state model, the putative intermediate state displays a slightly more favorable RSR value of 0.10 over those of the two end states each of 0.11. The mFo-DFc residual electron densities of refinement with all three strategies display weak positive and negative peaks adjacent to the pterin and γ-carboxylate groups of the ligands as evidenced at the ±3 σ level but little residual densities at the ±3.5 σ level (Fig. S1, supplementary material). This further indicates in real space that single state and multiple states can both fit the ligand electron densities with little residual electron densities remaining to suggest other alternative states. However, a closer examination of the negative electron density peaks near the pterin ring of the modeled putative intermediate facing the dihydrofolate end state suggests the absence of the latter. (Fig. S1, top panel, supplementary material). In addition, the 2mFo-DFc difference maps indicate that the benzoyl ring moiety of the initial tetrahydrofolate state in the two-state or three state models partially resides outside the electron density envelope. This suggests that atomic shifts occur from the initial state to a putative intermediate state, and the observed ligand electron densities cannot be attributed solely to a simple linear combination of the two end states (Fig. S2, supplementary material). In real space, the apparently better fit of the putative intermediate state over the two end state model is even clearer from the 2mFo-DFc maps at a higher cutoff, 1.5 σ level (Fig. S2, supplementary material, right panels). The final refined occupancy values of the two-state model are 0.47 and 0.45 for tetrahydrofolate and dihydrofolate, respectively. This suggests that the diffraction data can also be fit with approximately equal populations of two end ligand states in the reciprocal space (Table I), although the real space omit electron density suggests the presence of a putative intermediate ligand conformational state (Fig. 1). If the ligand and protein conformations are indeed coupled as we postulated earlier, then the real space electron density of the anchor residues of the partially disordered Met20 loop also favors a quasi-intermediate state rather than a simple linear combination of equal populations of the two end states (Fig. 3). The refined occupancy of the three-state model suggests that the most populated intermediate state (occupancy value of 0.45) that is approximately the sum of the two end states (occupancy values of 0.23 and 0.23). We then intentionally lower the initial occupancy of the putative intermediate state (0.1) with both the end states having higher occupancies (0.45 each). The refined occupancy is rather insensitive to the initial occupancy with a refined value of 0.47 for the intermediate state and refined values of 0.21 and 0.22 for tetrahydrofolate and dihydrofolate, respectively. To reflect an unbiased treatment, we deposit the three-state model in the Protein Data Bank (PDB, www.pdb.org) with the lower initial occupancy of the intermediate. Regardless of the overall number of states, the near equal populations of the two end states with that of the intermediate, when included, suggest that a mid-time point intermediate was captured along the decay time course.

### B. Fo-Fo isomorphous difference map

**FIG. 4.**
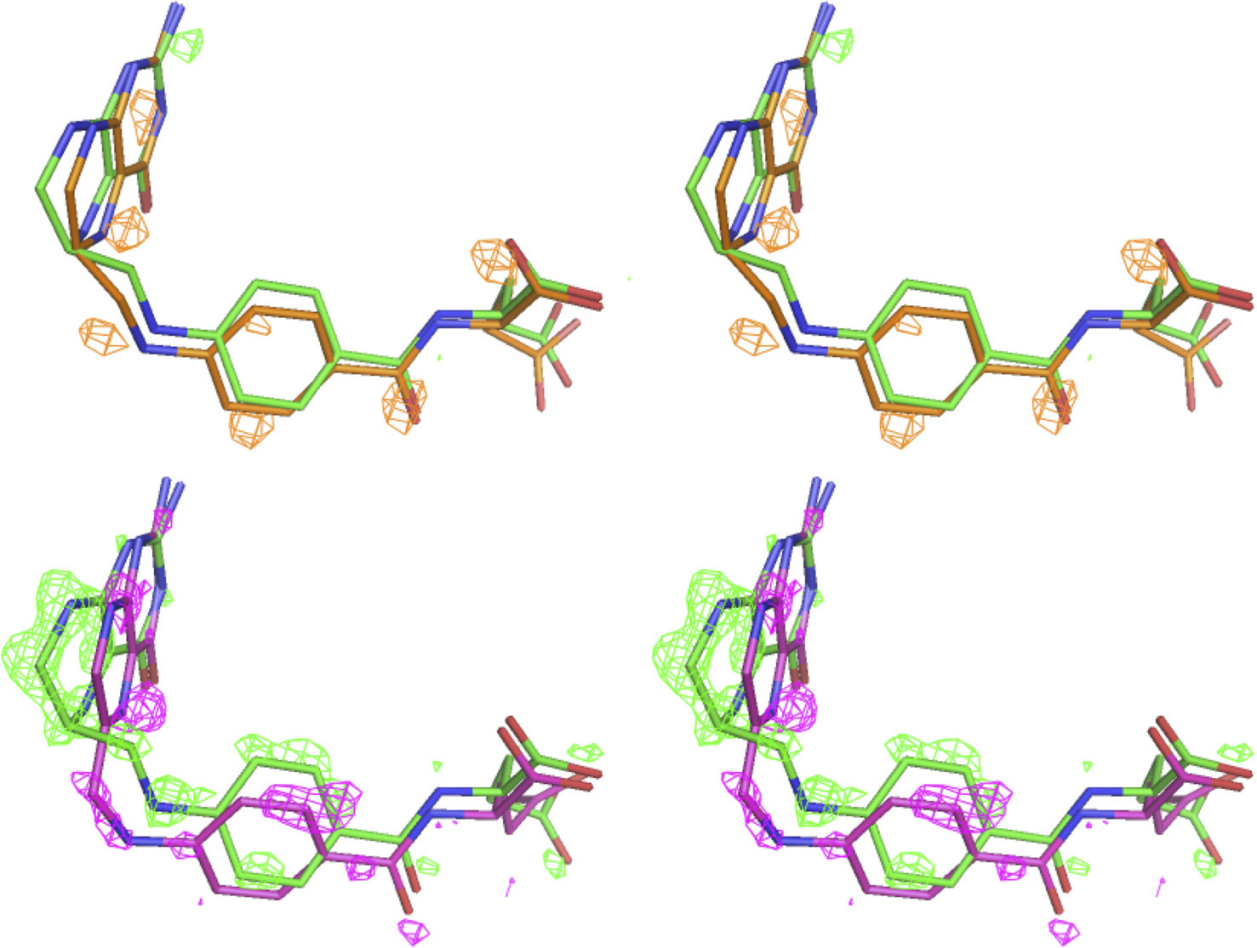
Fo-Fo isomorphous difference map in stereo views. Fo-Fo omit maps at ±3 σ are calculated with PHENIX^19^ for the pairwise comparison between tetrahydrofolate and intermediate (top panel) as well as between tetrahydrofolate and dihydrofolate (bottom panel). Color codes for carbon are tetrahydrofolate in green, intermediate in orange, and dihydrofolate in magenta. The left and right images are stereo views.

The difference map method has been often used for time-resolved crystallography to analyze the direction of atom shifts and intermediate states.^3,6,8–10,13^ We also performed a Fo-Fo difference map analysis using the ligand free model as unbiased phase input (PDB ID: 6CW7)^14^ to compare the intermediate dataset and the initial state tetrahydrofolate complex dataset (Fig. 4). The difference map shows positive Fo-Fo electron density near the atoms of the pterin, methylene linker, aminobenzoyl moiety, and α-carboxylate, in the direction shifted away from the initial tetrahydrofolate state, but hardly any negative electron density except near the exocyclic amino group of the pterin. It is known from other studies that the Fo-Fo difference electron densities rarely overlap exactly with the atom positions depending on the extent of shift.^3,6,8–10,13,24,25^ We further analyzed the Fo-Fo map to compare the two end-state datasets as a positive control (Fig. 4). There are clearly both positive and negative electron densities to suggest the completion of the reverse decay reaction from tetrahydrofolate to dihydrofolate. This indicates that the ligand atoms at the intermediate time point have a finite shift in the direction of decay that is small enough to differentiate it from the tetrahydrofolate and dihydrofolate beginning and end states. Importantly, the intuitive Fo-Fo difference map shown here qualitatively indicates the direction of the atom shifts rather than quantitatively estimating the percentage populations of different states or how each atom shifts. In order to assess the relatively small atom shifts of the intermediate state, we adopted the following Bayesian difference refinement approach.

### C. Bayesian difference refinement based on dataset pairs

Difference refinement aims to minimize the residual between the observed and calculated differences in the structure-factor amplitudes between two structures. The method was pioneered by Terwilliger and Berendzen^22,26^ and relies on the correlation of model errors between two similar datasets having very similar structures with relatively small atom shifts or conformational changes. The advantage of the Bayesian difference refinement^22^ method is that it accounts for correlated and uncorrelated model errors as well as the experimental uncertainty of each structure by introducing appropriate weighting terms. As described in Sec. II, ultimately a pseudo dataset was generated for refinement.^22^ The successful application of the method requires that the native structure be confidently determined with high accuracy, so that the very small shifts in the variant structure can be reliably estimated. This is suitable to the current case where the native tetrahydrofolate complex at 2 days of crystal growth was previously determined at a high resolution of 1.03 Å.^14^ Also as shown earlier, the isomorphous variant dataset at 3 days of crystal growth suggests finite but small changes based on the mFo-DFc omit maps (Fig. 2) and Fo-Fo difference maps (Fig. 4).

**FIG. 5.**
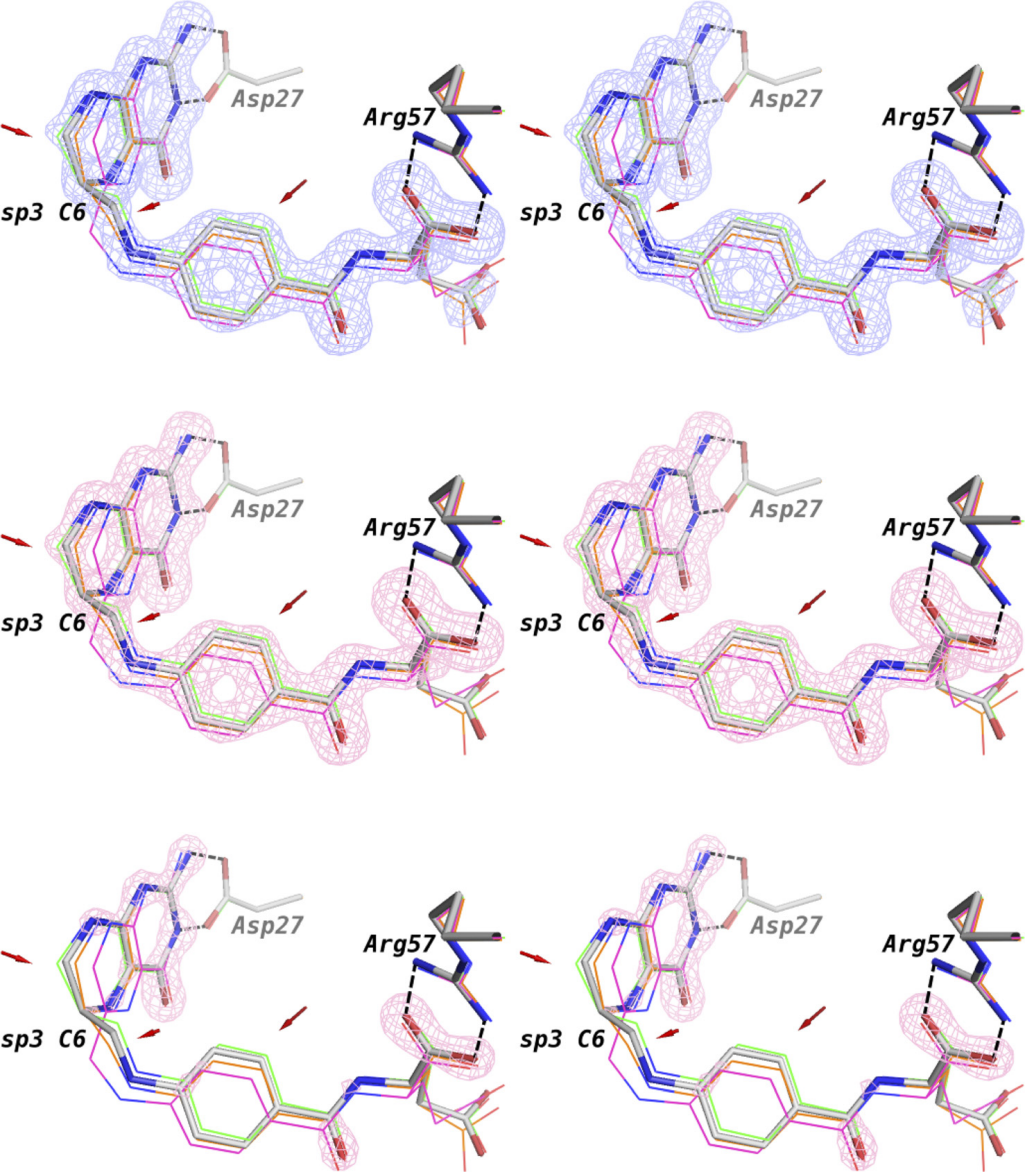
Bayesian difference refinement detects subtle atomic shifts from the initial state in stereo views. *F*_diff_ from the Bayesian weighted pseudo dataset was used as input in the same way as the regular *F*_obs_ for the calculation of electron density maps for Bayesian difference refinement as described in Sec. II. Top panel, 2mFo-DFc omit map at 1 σ (carve = 1.3); middle panel, mFo-DFc omit map at 6 σ; bottom panel, mFo-DFc omit map at 10 σ. Both the native (2 days' crystal) and variant (3 days' crystal) states use the same ligand geometry restraints (PDB ligand ID: THG for tetrahydrofolate). The Bayesian difference refinement ligand model is shown as sticks (carbon in grey). The initial state tetrahydrofolate (green), end state dihydrofolate (magenta), and the individually refined putative intermediate state (orange) are shown as thin lines and superposed based on protein coordinates for comparison. The guanidine and α-carboxylate groups form bidentate salt-bridges with Asp27 and Arg58, respectively (indicated by dashed lines). They display minimal shifts compared to the rest of pterin, methylene linker, and benzoyl moieties (general direction of shifts indicated by red arrows). Importantly, chemical labile C6 maintains *sp*^3^ tetrahedral geometry with little shift between 3 day and 2 day complexes but shows a relatively large shift for the dihydrofolate end state. The left and right images are stereo views.

The results of Bayesian difference refinement based on the paired datasets (2 days vs. 3 days of crystal growth) are summarized in Fig. 5 and Table II. The Bayesian difference refined structure displays ligand atoms lying somewhere in between the two end states, similar to the individually refined putative intermediate state (Fig. 5). However, it more closely resembles the initial tetrahydrofolate complex. This is expected, since the Bayesian difference refinement will bring model bias from the native structure as described by the method developer.^22^ Consequently, the method is very sensitive to any small true differences from the native structure, which is of interest here and in many similar cases.^22^ The overall trend of the atom shifts is in the same direction toward the formation of dihydrofolate as observed in the individual structure refinement (Fig. 5). The Bayesian difference refinement also suggests that the pterin ring becomes less puckered and thus favors charge delocalization at the intermediate time point, although the chemical nature of the ligand still appears reduced as the C6 position displays a tetrahedral geometry (as indicated by an arrow in Fig. 5). The quantitative estimation of atomic shifts is summarized in Table II which compares the different ligand states along the time course of decay of the tetrahydrofolate complex for the different refinement methods. Based on the RMSD shifts, it appears that the Bayesian difference refined structure is slightly more similar to the putative intermediate state than the native tetrahydrofolate state in the pterin moiety (0.14 vs. 0.18 Å), despite the overall similarity to the latter (0.32 vs 0.20 Å) as expected due to the introduction of native model bias (last two columns of Table II). This again supports the possible presence of a quasi-stable intermediate state along the reaction coordinate of the current slow reverse oxidation process that favors charge delocalization through a near planar pterin ring, followed by a rate-limiting C6-H breaking step to form the end point oxidized dihydrofolate state. A geometrically similar and relatively stable quinonoid intermediate has been experimentally observed before for the oxidation of a closely related compound tetrahydropterin in solution by polarography and UV-vis spectroscopy.^27,28^ This quinonoid intermediate was formed through proton coupled electron transfer under either anaerobic conditions in the presence of ferricyanide or aerobic conditions.^27,28^ However, the oxidation of free tetrahydrofolate in solution generated 6,8-dihydrofolate anaerobically^29^ or pterins aerobically.^30^ However, a quinonoid intermediate was never experimentally observed but nonetheless proposed as a transient unstable state following the same oxidation mechanism of tetrahydropterin.^29,30^

**TABLE II.** Shifts (Å) of ligand non-hydrogen atom positions between superposed protein structures.^a^

Ligand atoms	FH4 to Intermediate	FH4 to FH2	FH4 to Bayesian	Bayesian to Intermediate
***Pterin RMSD***	**0.30**	**0.58**	**0.18**	**0.14**
N1	0.11	0.21	0.10	0.06
C2	0.05	0.13	0.07	0.07
N2	0.18	0.40	0.09	0.13
N3	0.09	0.19	0.09	0.08
C4	0.10	0.17	0.13	0.04
O4	0.10	0.15	0.14	0.04
C4a	0.25	0.48	0.19	0.09
N5	0.41	0.82	0.25	0.19
C6	0.16	0.69	0.09	0.09
C7	0.66	1.18	0.34	0.32
N8	0.46	0.83	0.25	0.21
C8a	0.26	0.49	0.16	0.12
***Methylene-p-aminobenzoyl RMSD***	**0.59**	**0.97**	**0.24**	**0.39**
C9	0.66	1.29	0.29	0.38
N10	0.55	1.00	0.28	0.27
C1'	0.34	0.70	0.18	0.20
C2'	0.75	0.89	0.24	0.56
C3'	0.83	1.03	0.29	0.58
C4'	0.49	0.84	0.25	0.25
C5'	0.71	1.18	0.25	0.50
C6'	0.72	1.22	0.25	0.50
C11	0.25	0.67	0.16	0.14
O11	0.21	0.65	0.13	0.14
***Glutamate tail RMSD***	**0.46**	**1.01**	**0.17**	**0.39**
N	0.25	0.66	0.17	0.13
Cα	0.26	0.69	0.18	0.14
Cβ	0.26	0.76	0.17	0.13
Cγ	0.24	0.91	0.16	0.11
Cδ	0.49	0.40	0.13	0.43
Oε1	0.87	1.23	0.28	0.78
Oε2	0.88	2.38	0.16	0.76
C	0.22	0.49	0.13	0.17
OX1	0.22	0.43	0.11	0.20
OX2	0.15	0.38	0.12	0.17
***All-atom RMSD***	**0.46**	**0.86**	**0.20**	**0.32**

^a^ All structures were aligned based on protein coordinates using the “LSQ superpose” function of COOT.^18^ The structures include the FH4 or tetrahydrofolate complex (PDB: 6CW7), FH2 or dihydrofolate complex (PDB: 6CXK), intermediate complex (single-state model in the current study), and Bayesian difference refinement complex model.

Due to the finite coupling of ligand and protein conformational changes and the limited protein motion in crystals, our working hypothesis is that the crystalline protein bound tetrahydrofolate is oxidized at a slower overall rate as compared to solution, where the C6-H bond breaking step (*sp*^3^ to *sp*^2^ C6 major geometry change) becomes at least partially rate-limiting. As previously observed, the guanidine and the α-carboxylic groups at two ends of the ligand are anchored by two strong bidentate salt-bridges to Asp27 and Arg57 in *E. coli* DHFR (Fig. 5).^14^ This is also evidenced here by the clear mFo-DFc omit electron densities of the pairwise Bayesian difference refinement traceable even at 10 σ (Fig. 5) and minimal atomic shifts compared to the rest of the ligand during the decay (Fig. 5 and N1, C2, N2, N3; OX1, OX2, C in Table II). Importantly, these pairs of salt-bridging residues are highly conserved in all DHFRs. Consequently. there will be limited conformational space to explore for the enzyme-bound ligand as compared to free solution. This would necessitate the twisting motion of the ligand in both the catalytic forward (with NADPH) and the slow reverse (absence of NADPH) reactions involving the conversion between the *sp*^3^ and *sp*^2^ geometry at C6 and the concomitant rotation of the aminobenzoyl group connected by the methylene linkage. In recognition of the essential structural changes between physiologically relevant ligand states, we previously proposed the design of slow-onset inhibitors to closely mimic the binding pose of tetrahydrofolate rather than general active site blockers that are more susceptible to mutation based emergence of drug resistance.^14,15,31,32^

## IV. CONCLUSIONS

We report here a critical assessment of the ligand identity during a slow reverse oxidative decay of tetrahydrofolate in the enzyme bound crystalline form. The linearly combined multi-state analysis suggests that we captured a putative intermediate state featuring a more planar pterin maintaining the *sp*^3^ C6 geometry based on both the individual refinement of a single dataset and Bayesian difference refinement using paired datasets. A geometrically similar quinonoid intermediate was experimentally observed for the oxidation of related tetrahydropterin^27,28^ and proposed for the oxidation of tetrahydrofolate free in solution;^29,30^ both occur on the minute time scale.^28,33^ This implies that the extension of the conjugation system in the bicyclic pterin favoring charge delocalization may occur either stably or transiently depending on the exocyclic derivatives at the C6 position during the rate-limiting C6-H bond breaking step. Here, the overall reverse oxidation of tetrahydrofolate is slowed down to an appreciable extent due to the coupling of ligand and protein conformational changes and the limited protein motion in the crystalline state. As shown in Fig. 3, one end of the intermediate is basically clamped by the protein in the same conformation as that in the tetrahydrofolate structure, while the other more mobile end adopts a conformation much closer to the dihydrofolate oxidation product. Time-resolved crystallography is achieved due to a relatively uniform starting point, the rate-limiting product release complex of *E. coli* DHFR, whose irreversible oxidation was likely triggered by a finite amount of freely diffusing oxygen in the generally aerobic crystallization conditions, despite the presence of the mM level of the reducing agent in the crystallization drop. The limitation of the current method in delineating the exact ligand populations and chemical nature can be complemented by orthogonal approaches such as time-resolved spectroscopy at controlled oxidant levels. Such complementary approaches might provide additional evidence to allow further differentiation between the presence of the putative intermediate state and a spatially averaged mixture of two end states only. The capability of the current method to exhibit time-resolved crystallography on a slow time scale may inspire its application to other systems via coupling chemistry with protein motions to probe substantial ligand chemical changes without the need for a chemical modification to the native system. The key is that the protein itself needs to at least partially constrain the ligand motion so that a trapped intermediate ligand state results. The generality of such an approach needs to be explored but a possible place to start is with slow-onset tight binders that are subject to oxidative decay on product release. Unresolved questions are whether these observation are physiologically relevant, and in particular, in cells, how rapid is tetrahydrofolate consumed for the production of DNA base pairs relative to its decay to dihydrofolate, which in free solution under aerobic conditions occurs on the order of minutes. These issues will be explored in future work.

## SUPPLEMENTARY MATERIAL

See supplementary material for Figs. S1 and S2 on electron densities of single-state and multi-state refinements and also a supplementary coordinate file containing multiple-aligned PDB entries of *E. coli* DHFR deposited by our group to facilitate comparison and use of these structures. In particular, 6MR9, 6MT8, 6MTH, 6CXK, 6CYV, and 6CQA were superposed with PyMOL onto the published FH4 complex (6CW7).^14^

## References

[1] H. van den Bedem and J. S. Fraser , “Integrative, dynamic structural biology at atomic resolution-it's about time,” Nat. Methods 12, 307–318 (2015). 10.1038/nmeth.3324 25825836 PMC4457290

[2] F. Schotte , M. Lim , T. A. Jackson , A. V. Smirnov , J. Soman , J. S. Olson , G. N. Phillips, Jr. , M. Wulff , and P. A. Anfinrud , “Watching a protein as it functions with 150-ps time-resolved x-ray crystallography,” Science 300, 1944–1947 (2003). 10.1126/science.1078797 12817148

[3] J. E. Knapp , R. Pahl , V. Srajer , and W. E. Royer, Jr. , “Allosteric action in real time: Time-resolved crystallographic studies of a cooperative dimeric hemoglobin,” Proc. Natl. Acad. Sci. U. S. A. 103, 7649–7654 (2006). 10.1073/pnas.0509411103 16684887 PMC1472499

[4] F. Schotte , H. S. Cho , V. R. I. Kaila , H. Kamikubo , N. Dashdorj , E. R. Henry , T. J. Graber , R. Henning , M. Wulff , G. Hummer , M. Kataoka , and P. A. Anfinrud , “Watching a signaling protein function in real time via 100-ps time-resolved Laue crystallography,” Proc. Natl. Acad. Sci. U. S. A. 109, 19256–19261 (2012). 10.1073/pnas.1210938109 23132943 PMC3511082

[5] Y. O. Jung , J. H. Lee , J. Kim , M. Schmidt , K. Moffat , V. Srajer , and H. Ihee , “Volume-conserving trans-cis isomerization pathways in photoactive yellow protein visualized by picosecond X-ray crystallography,” Nat. Chem. 5, 212–220 (2013). 10.1038/nchem.1565 23422563 PMC3579544

[6] J. Tenboer , S. Basu , N. Zatsepin , K. Pande , D. Milathianaki , M. Frank , M. Hunter , S. Boutet , G. J. Williams , J. E. Koglin , D. Oberthuer , M. Heymann , C. Kupitz , C. Conrad , J. Coe , S. Roy-Chowdhury , U. Weierstall , D. James , D. Wang , T. Grant , A. Barty , O. Yefanov , J. Scales , C. Gati , C. Seuring , V. Srajer , R. Henning , P. Schwander , R. Fromme , A. Ourmazd , K. Moffat , J. J. Van Thor , J. C. H. Spence , P. Fromme , H. N. Chapman , and M. Schmidt , “Time-resolved serial crystallography captures high-resolution intermediates of photoactive yellow protein,” Science 346, 1242–1246 (2014). 10.1126/science.1259357 25477465 PMC4361027

[7] C. Kupitz , S. Basu , I. Grotjohann , R. Fromme , N. A. Zatsepin , K. N. Rendek , M. S. Hunter , R. L. Shoeman , T. A. White , D. Wang , D. James , J. H. Yang , D. E. Cobb , B. Reeder , R. G. Sierra , H. Liu , A. Barty , A. L. Aquila , D. Deponte , R. A. Kirian , S. Bari , J. J. Bergkamp , K. R. Beyerlein , M. J. Bogan , C. Caleman , T. C. Chao , C. E. Conrad , K. M. Davis , H. Fleckenstein , L. Galli , S. P. Hau-Riege , S. Kassemeyer , H. Laksmono , M. Liang , L. Lomb , S. Marchesini , A. V. Martin , M. Messerschmidt , D. Milathianaki , K. Nass , A. Ros , S. Roy-Chowdhury , K. Schmidt , M. Seibert , J. Steinbrener , F. Stellato , L. Yan , C. Yoon , T. A. Moore , A. L. Moore , Y. Pushkar , G. J. Williams , S. Boutet , R. B. Doak , U. Weierstall , M. Frank , H. N. Chapman , J. C. H. Spence , and P. Fromme , “Serial time-resolved crystallography of photosystem II using a femtosecond X-ray laser,” Nature 513, 261–265 (2014). 10.1038/nature13453 25043005 PMC4821544

[8] J. Kern , R. Tran , R. Alonso-Mori , S. Koroidov , N. Echols , J. Hattne , M. Ibrahim , S. Gul , H. Laksmono , R. G. Sierra , R. J. Gildea , G. Han , J. Hellmich , B. Lassalle-Kaiser , R. Chatterjee , A. S. Brewster , C. A. Stan , C. Glockner , A. Lampe , D. DiFiore , D. Milathianaki , A. R. Fry , M. M. Seibert , J. E. Koglin , E. Gallo , J. Uhlig , D. Sokaras , T. C. Weng , P. H. Zwart , D. E. Skinner , M. J. Bogan , M. Messerschmidt , P. Glatzel , G. J. Williams , S. Boutet , P. D. Adams , A. Zouni , J. Messinger , N. K. Sauter , U. Bergmann , J. Yano , and V. K. Yachandra , “Taking snapshots of photosynthetic water oxidation using femtosecond X-ray diffraction and spectroscopy,” Nat. Commun. 5, 4371 (2014). 10.1038/ncomms5371 25006873 PMC4151126

[9] M. Suga , F. Akita , M. Sugahara , M. Kubo , Y. Nakajima , T. Nakane , K. Yamashita , Y. Umena , M. Nakabayashi , T. Yamane , T. Nakano , M. Suzuki , T. Masuda , S. Inoue , T. Kimura , T. Nomura , S. Yonekura , L. J. Yu , T. Sakamoto , T. Motomura , J. H. Chen , Y. Kao , T. Noguchi , K. Tono , Y. Joti , T. Kameshima , T. Hatsui , E. Nango , R. Tanaka , H. Naitow , Y. Matuura , A. Yamashita , M. Yamamoto , O. Nureki , M. Yabashi , T. Ishikawa , S. Iwata , and J. R. Shen , “Light-induced structural changes and the site of O=O bond formation in PSII caught by XFEL,” Nature 543, 131–135 (2017). 10.1038/nature21400 28219079

[10] P. Nogly , V. Panneels , G. Nelson , C. Gati , T. Kimura , C. Milne , D. Milathianaki , M. Kubo , W. Wu , C. Conrad , J. Coe , R. Bean , Y. Zhao , P. Bath , R. Dods , R. Harimoorthy , K. R. Beyerlein , J. Rheinberger , D. James , D. DePonte , C. Li , L. Sala , G. J. Williams , M. S. Hunter , J. E. Koglin , P. Berntsen , E. Nango , S. Iwata , H. N. Chapman , P. Fromme , M. Frank , R. Abela , S. Boutet , A. Barty , T. A. White , U. Weierstall , J. Spence , R. Neutze , G. Schertler , and J. Standfuss , “Lipidic cubic phase injector is a viable crystal delivery system for time-resolved serial crystallography,” Nat. Commun. 7, 12314 (2016). 10.1038/ncomms12314 27545823 PMC4996941

[11] C. Kupitz , J. L. Olmos, Jr. , M. Holl , L. Tremblay , K. Pande , S. Pandey , D. Oberthur , M. Hunter , M. Liang , A. Aquila , J. Tenboer , G. Calvey , A. Katz , Y. Chen , M. O. Wiedorn , J. Knoska , A. Meents , V. Majriani , T. Norwood , I. Poudyal , T. Grant , M. D. Miller , W. Xu , A. Tolstikova , A. Morgan , M. Metz , J. M. Martin-Garcia , J. M. Zook , S. Roy-Chowdhury , J. Coe , N. Nagaratnam , D. Meza , R. Fromme , S. Båasu , M. Frank , T. White , A. Barty , S. Bajt , O. Yefanov , H. N. Chapman , N. Zatsepin , G. Nelson , U. Weierstall , J. Spence , P. Schwander , L. Pollack , P. fromme , A. Ourmazd , G. N. Phillips, Jr. , and M. Schmidt , “Structural enzymology using X-ray free electron lasers,” Struct. Dyn. 4, 044003 (2017). 10.1063/1.4972069 28083542 PMC5178802

[12] J. L. Olmos, Jr. , S. Pandey , J. M. Martin-Garcia , G. Calvey , A. Katz , J. Knoska , C. Kupitz , M. S. Hunter , M. Liang , D. Oberthuer , O. Yefanov , M. Wiedorn , M. Heyman , M. Holl , K. Pande , A. Barty , M. D. Miller , S. Stern , S. Roy-Chowdhury , J. Coe , N. Nagaratnam , J. Zook , J. Verburgt , T. Norwood , I. Poudyal , D. Wu , J. Koglin , M. H. Seaberg , Y. Zhao , S. Bajt , T. Grant , V. Mariani , G. Nelson , G. Subramanian , E. Bae , R. Fromme , R. Fung , P. Schwander , M. Frank , T. A. White , U. Weierstall , N. Zatsepin , J. Spence , P. Fromme , H. N. Chapman , L. Pollack , L. Tremblay , A. Ourmazd , G. N. Phillips, Jr. , and M. Schmidt , “Enzyme intermediates captured “on the fly” by mix-and-inject serial crystallography,” BMC Biol. 16, 59 (2018). 10.1186/s12915-018-0524-5 29848358 PMC5977757

[13] J. R. Stagno , Y. Liu , Y. R. Bhandari , C. E. Conrad , S. Panja , M. Swain , L. Fan , G. Nelson , C. Li , D. R. Wendel , T. A. White , J. D. Coe , M. O. Wiedorn , J. Knoska , D. Oberthuer , R. A. Tuckey , P. Yu , M. Dyba , S. G. Tarasov , U. Weierstall , T. D. Grant , C. D. Schwieters , J. Zhang , A. R. Ferre-D'Amare , P. Fromme , D. E. Draper , M. Liang , M. S. Hunter , S. Boutet , K. Tan , X. Zuo , X. Ji , A. Barty , N. A. Zatsepin , H. N. Chapman , J. C. Spence , S. A. Woodson , and Y. X. Wang , “Structures of riboswitch RNA reaction states by mix-and-inject XFEL serial crystallography,” Nature 541, 242–246 (2017). 10.1038/nature20599 27841871 PMC5502819

[14] H. Cao , M. Gao , H. Zhou , and J. Skolnick , “The crystal structure of a tetrahydrofolate-bound dihydrofolate reductase reveals the origin of slow product release,” Commun. Biol. 1, 226 (2018). 10.1038/s42003-018-0236-y 30564747 PMC6290769

[15] J. V. Rodrigues , S. Bershtein , A. Li , E. R. Lozovsky , D. L. Hartl , and E. I. Shakhnovich , “Biophysical principles predict fitness landscapes of drug resistance,” Proc. Natl. Acad. Sci. U. S. A. 113, E1470–E1478 (2016). 10.1073/pnas.1601441113 26929328 PMC4801265

[16] W. Kabsch , “XDS,” Acta Crystallogr., Sec. D: Biol. Crystallogr. 66, 125–132 (2010). 10.1107/S0907444909047337 PMC2815665 20124692

[17] A. J. McCoy , R. W. Grosse-Kunstleve , P. D. Adams , M. D. Winn , L. C. Storoni , and R. J. Read , “Phaser crystallographic software,” J. Appl. Crystallogr. 40, 658–674 (2007). 10.1107/S0021889807021206 19461840 PMC2483472

[18] P. Emsley and K. Cowtan , “Coot: Model-building tools for molecular graphics,” Acta Crystallogr., Sec. D: Biol. Crystallogr. 60, 2126–2132 (2004). 10.1107/S0907444904019158 15572765

[19] P. D. Adams , P. V. Afonine , G. Bunkoczi , V. B. Chen , I. W. Davis , N. Echols , J. J. Headd , L. W. Hung , G. J. Kapral , R. W. Grosse-Kunstleve , A. J. McCoy , N. W. Moriarty , R. Oeffner , R. J. Read , D. C. Richardson , J. S. Richardson , T. C. Terwilliger , and P. H. Zwart , “PHENIX: A comprehensive Python-based system for macromolecular structure solution,” Acta Crystallogr., Sec. D: Biol. Crystallogr. 66, 213–221 (2010). 10.1107/S0907444909052925 PMC2815670 20124702

[20] V. B. Chen , W. B. Arendall, 3rd , J. J. Headd , D. A. Keedy , R. M. Immormino , G. J. Kapral , L. W. Murray , J. S. Richardson , and D. C. Richardson , “MolProbity: All-atom structure validation for macromolecular crystallography,” Acta Crystallogr., Sec. D: Biol. Crystallogr. 66, 12–21 (2010). 10.1107/S0907444909042073 PMC2803126 20057044

[21] The PyMOL Molecular Graphics System, version 1.3 Schrodinger, LLC, 2010.

[22] T. C. Terwilliger and J. Berendzen , “Bayesian difference refinement,” Acta Crystallogr., Sec. D: Biol. Crystallogr. 52, 1004–1011 (1996). 10.1107/S0907444996006725 15299610

[23] T. C. Terwilliger and J. Berendzen , “Automated MAD and MIR structure solution,” Acta Crystallogr., Sec. D: Biol. Crystallogr. 55, 849–861 (1999). 10.1107/S0907444999000839 PMC2746121 10089316

[24] M. A. Rould and C. W. Carter, Jr. , “Isomorphous difference methods,” Methods. Enzymol. 374, 145–163 (2003). 10.1016/S0076-6879(03)74007-5 14696372

[25] Z. Ren , P. W. Y. Chan , K. Moffat , E. F. Pai , W. E. Royer, Jr. , V. Srajer , and X. Yang , “Resolution of structural heterogeneity in dynamic crystallography,” Acta Crystallogr., Sec. D: Biol. Crystallogr. 69, 946–959 (2013). 10.1107/S0907444913003454 PMC3663119 23695239

[26] T. C. Terwilliger and J. Berendzen , “Difference refinement: Obtaining differences between two related structures,” Acta Crystallogr., Sec. D: Biol. Crystallogr. 51, 609–618 (1995). 10.1107/S0907444994013247 15299790

[27] D. J. Vonderschmitt and K. G. Scrimgeour , “Reaction of Cu2+ and Fe3+ with tetrahydropteridines,” Biochem. Biophys. Res. Commun. 28, 302–308 (1967). 10.1016/0006-291X(67)90309-9 6055158

[28] M. C. Archer and K. G. Scrimgeour , “Rearrangement of quinonoid dihydropteridines to 7,8-dihydropteridines,” Can. J. Biochem. 48, 278–287 (1970). 10.1139/o70-049 5438318

[29] D. Chippel and K. G. Scrimgeour , “Oxidative degradation of dihydrofolate and tetrahydrofolate,” Can. J. Biochem. 48, 999–1009 (1970). 10.1139/o70-156 5273718

[30] L. S. Reed and M. C. Archer , “Oxidation of tetrahydrofolic acid by air,” J. Agric. Food. Chem. 28, 801–805 (1980). 10.1021/jf60230a044

[31] E. Toprak , A. Veres , J. B. Michel , R. Chait , D. L. Hartl , and R. Kishony , “Evolutionary paths to antibiotic resistance under dynamically sustained drug stress,” Nat. Genet. 44, 101–105 (2012). 10.1038/ng.1034 PMC3534735 22179135

[32] B. Srinivasan , J. V. Rodrigues , S. Tonddast-Navaei , E. Shakhnovich , and J. Skolnick , “Rational design of novel allosteric dihydrofolate reductase inhibitors showing antibacterial effects on drug-resistant *Escherichia coli* escape variants,” ACS Chem. Biol. 12, 1848–1857 (2017). 10.1021/acschembio.7b00175 28525268 PMC5819740

[33] R. L. Blakley , “Spectrophotometric studies on the combination of formaldehyde with tetrahydropteroylglutamic acid and other hydropteridines,” Biochem. J. 74, 71–82 (1960). 10.1042/bj0740071 13801272 PMC1204051

[34] A. A. Vaguine , J. Richelle , and S. J. Wodak , “SFCHECK: A unified set of procedure for evaluating the quality of macromolecular structure-factor data and their agreement with atomic model,” Acta Crystallogr., Sec. D: Biol. Crystallogr. 55, 191–205 (1999). 10.1107/S0907444998006684 10089410

[35] M. D. Winn , C. C. Ballard , K. D. Cowtan , E. J. Dodson , P. Emsley , P. R. Evans , R. M. Keegan , E. B. Krissinel , A. G. Leslie , A. McCoy , S. J. McNicholas , G. N. Murshudov , N. S. Pannu , E. A. Potterton , H. R. Powell , R. J. Read , A. Vagin , and K. S. Wilson , “Overview of the CCP4 suite and current developments,” Acta Crystallogr., Sec. D: Biol. Crystallogr. 67, 235–242 (2011). 10.1107/S0907444910045749 PMC3069738 21460441

[36] P. A. Karplus and K. Diederichs , “Linking crystallographic model and data quality,” Science 336, 1030–1033 (2012). 10.1126/science.1218231 22628654 PMC3457925

